# Power contestation and regulation in digital platform ecosystems—The case of the EU’s Digital Markets Act

**DOI:** 10.1007/s12525-025-00858-9

**Published:** 2026-01-13

**Authors:** Victorine Verlooy, Vincent Heimburg, Maximilian Schreieck, Manuel Wiesche

**Affiliations:** 1 Department of Information Systems, Production and Logistics Management, University of Innsbruck, Universitätsstraße 15, 6020 Innsbruck, Austria; 2 Chair of Digital Transformation, TU Dortmund University, Otto-Hahn Str. 4, 44227 Dortmund, Germany

**Keywords:** Digital platform ecosystems, Platform power, Platform owners, Complementors, Digital Markets Act (DMA), Regulation, L500, M150

## Abstract

In this study, we investigate how power contestation between complementors and powerful platform owners unfolds in digital platform ecosystems as they come under regulatory scrutiny. Platform owners like Alphabet/Google, Apple, Amazon, and Meta/Facebook dominate their respective ecosystems, leveraging network effects and market power to maintain their positions, giving them a powerful advantage vis-à-vis supply-side complementors. Complementors have become increasingly dependent on these platforms and struggle due to their weakened position. In the EU, the European Commission has implemented the Digital Markets Act, which aims to increase fairness and contestability within and across digital platform ecosystems. Using a case study approach, we analyze how regulatory scrutiny, such as the Digital Markets Act, has affected power contestation between platform owners and complementors. In particular, we investigate app store and search platform ecosystems. We establish a conceptual framework of power contestation that shows how complementors and platform owners interact directly and through the regulator as a mediator, vying for power in the ecosystem. We thereby contribute to the emerging Information Systems literature on power dynamics and the regulation of digital platform ecosystems.

**Supplementary Information:**

The online version contains supplementary material available at 10.1007/s12525-025-00858-9.

## Introduction

In many digital platform ecosystems, powerful platform owners such as Alphabet (Google), Apple, Amazon, and Meta (Facebook) hold significant power over platform complementors, including third-party developers and sellers (Lv & Schotter, 2024). The platform owners’ power in the ecosystem directly stems from dominance in their target markets, driven by network effects: as user bases grow, they attract more complementors, and vice versa (Kim et al., 2016). Consequently, these markets exhibit “winner-takes-all” dynamics, leading to one or sometimes two digital platform owners emerging to dominate the market (Evans & Schmalensee, 2007; Zhu & Iansiti, 2012). Complementors joining the ecosystems of these dominant platform owners, therefore, have a pronounced power disadvantage. While these power dynamics benefit powerful platform owners, they create challenges for complementors, who face shrinking revenue shares and reduced decision-making freedom as platforms become more powerful (Soper, 2023). This imbalance can undermine innovation, increase costs for consumers, and ultimately harm consumer welfare (Nambisan & Baron, 2021; Subcommittee on Antitrust, 2020).

This growing power imbalance has prompted regulatory scrutiny, mainly through initiatives such as the European Union’s (EU) Digital Markets Act (DMA) and the UK’s Digital Markets, Competition and Consumers Act (DMCCA). The DMA targets powerful platform owners—designated as “gatekeepers”—aiming to ensure fairness and contestability within and across ecosystems (EU, 2022; European Commission, 2024; Heimburg & Wiesche, 2022). Unlike traditional antitrust frameworks, which address anti-competitive behavior after it occurs, the DMA adopts an ex ante approach, proactively imposing obligations to prevent abuses of market power. Such measures have attracted significant attention, as current antitrust laws often fail to address the structural dependencies and technological lock-ins that reinforce the disadvantages of complementors (Lv & Schotter, 2024).

The DMA provides a unique and timely opportunity to explore how regulatory scrutiny can affect power dynamics in digital platform ecosystems. By focusing on the developmental phase of the DMA, we examine how power is actively negotiated, rather than merely exercised, as key stakeholders seek to shape the framing, scope, and content of the regulatory text. The developmental phase of regulation is crucial, as the details are still in flux and prone to lobbying efforts by other stakeholders, and the outcome will shape regulation for years to come.

Due to their remarkable success, digital platform ecosystems have become a central topic in Information Systems (IS) research. Most studies focus on the architecture and governance of digital platform ecosystems (de Reuver, Nederstigt, et al., 2018; Tiwana, 2014); however, the power dynamics between platform owners and complementors have begun to attract IS researchers’ interest (e.g., Gleiss et al., 2023; Heimburg et al., 2024; Hurni et al., 2022). Scholars call for critically examining platform governance mechanisms and assessing their role in addressing power issues (Cusumano, 2022; de Reuver, Sørensen, et al., 2018a, 2018b; Gawer, 2021a). The DMA’s design and implementation provide a fertile setting to study these power dynamics under regulatory scrutiny, as it represents a landmark intervention aimed at rebalancing power in digital markets.

We thus pose the following research question: *How does regulatory scrutiny, as exemplified by the EU’s Digital Markets Act, affect the power dynamics in digital platform ecosystems dominated by a powerful platform owner?*

To address this research question, we conduct a revelatory case study on the EU’s DMA—a qualitative research approach that investigates a contemporary phenomenon in depth and within its real-world context (Yin, 2014). This previously inaccessible case offers a unique opportunity to examine previously unexplored power dynamics during its regulatory development phase. The DMA is particularly relevant because it targets dominant platform owners across multiple ecosystems, imposing obligations to ensure fairness and contestability, intended to limit their power. By focusing on the developmental phase of the DMA (June 2020 to March 2024), we analyze how platform owners, complementors, and the EU, as the regulatory body, engaged in discursive power struggles to shape the regulation. Our dataset comprises 417 documents and three videos, totaling 23 hours of official European Commission workshops on the DMA, selected for in-depth analysis and coding. Our paper focuses on two ecosystems—app stores and search platforms—with Apple and Alphabet (Google) as designated gatekeepers. These ecosystems are particularly illustrative of the power asymmetries the DMA seeks to address, as they are characterized by entrenched dominance and significant complementor dependency.

We contribute to understanding power dynamics in digital platform ecosystems by developing a conceptual framework and propositions that explain power contestation, that is, how power is negotiated and redefined between platform owners and complementors under regulatory scrutiny. This framework highlights the role of the regulator as a mediator, positioned between competing claims and pressures from platform owners and complementors. Our work sheds light on the mechanisms used by platform owners to resist regulatory constraints and protect their dominant position, as well as the strategies complementors use to influence regulators and advocate for a rebalance of power. This results in a cycle of tension in which power is continuously contested. By examining these dynamics in the context of regulatory oversight, we reveal that power contestation is a recurring cycle: platform owners defend the status quo, while complementors push for a redistribution of power.

## Theoretical background

To explore power dynamics in digital platform ecosystems under regulatory scrutiny, it is essential to first understand the underlying factors that shape them. This includes examining the roles of platform owners, complementors, and end users, as well as the governance mechanisms that influence their interactions.

### Digital platforms and ecosystems

Digital platforms connect complementors—who contribute applications, services, and other products—to platform owners, who manage and control the digital platform (Eisenmann et al., 2009; Ramasundaram et al., 2023). These interactions form digital platform ecosystems (Heimburg & Wiesche, 2022; Hein et al., 2020; Ramasundaram et al., 2023). Examples of digital platforms include mobile operating systems like Android (Google) and iOS (Apple), where platform owners provide infrastructure and access, while third-party developers build complementary apps, creating a mutually attractive ecosystem (Tafesse, 2021).

Scholars distinguish between innovation platforms, which enable complementors to develop modules, transaction platforms, which facilitate transactions between buyers and sellers (Gawer & Bonina, 2024), and hybrid platforms combining both aspects, such as iOS with app development and the Apple App Store for distribution (Bonina et al., 2021; Cusumano et al., 2019).

IS, management, and economics scholars have studied digital platforms intensely, focusing on platform openness, governance (e.g., the design of boundary resources), and value creation and capture. These strands focus on designing value exchanges between the platform owner and complementors on a technical, organizational, or economic level (Easley et al., 2018; Huber et al., 2017; Kapoor et al., 2021; Wareham et al., 2015). Openness fosters complementor participation and generativity but can limit platform owner control, leading to security and competition risks (Benlian et al., 2015; Hein et al., 2020; Karhu et al., 2018; Ondrus et al., 2015). Platform owners use boundary resources (e.g., application programming interfaces, software development kits, documentation) to cultivate their ecosystem. Specifically, boundary resources transfer capability to complementors while they help the platform owner maintain control over the platform (Ghazawneh & Henfridsson, 2013; Schreieck et al., 2022). Thus, boundary resources can enhance value co-creation and ecosystem value (Tan et al., 2020) but are mainly designed by platform owners, though third-party influence is possible with permission (Eaton et al., 2015).

To create a flourishing platform ecosystem, platform owners must balance enabling value co-creation with capturing enough value themselves (Gawer, 2021b; Schreieck et al., 2021; Tiwana et al., 2010). New platforms should first strive to increase network effects and complementor value co-creation and only start capturing value once they have established a large and vibrant platform ecosystem (Parker et al., 2016; Tan et al., 2016).

Over the years, dominant platform owners have emerged in domains such as mobile operating systems (Android, iOS), e-commerce (Amazon), and social media (Facebook, Instagram), driven by “winner-takes-all” dynamics (Evans & Schmalensee, 2007; Zhu & Iansiti, 2012). As a result, platform owners gain power as complementors lack viable alternatives, raising governance and regulatory concerns (Gawer, 2021a). Therefore, IS scholars have started to increasingly study power distribution and regulatory impacts in platform ecosystems.

### Platform power, power dynamics, and regulation

In digital platform ecosystems, power is multi-dimensional and dynamic, unfolding through evolving relationships between platform owners and complementors (Simeonova et al., 2021). Platform owners typically have strong decision-making power, yet transfer some decision rights to complementors by implementing appropriate governance mechanisms to incentivize complementors’ voluntary contributions to the ecosystem (Tiwana et al., 2010). These interactions can be understood through the lens of power dynamics, which refer to the complex and evolving relationships through which power is distributed and exercised among actors, systems, and technologies (Hurni et al., 2022; Simeonova et al., 2021). Power dynamics are shaped by market structures, bargaining positions, and platform governance, and take forms from coercion to empowerment and hierarchical transparency (Fleming & Spicer, 2014; Simeonova et al., 2021).

Empirical studies illustrate these dynamics. Perrons (2009) shows how platform leaders balance trust and coercion to maintain control, often disguising power as collaboration, exposing complementors to risk. Tiwana et al. (2010) highlight tensions between owner control and developer autonomy, while Eaton et al. (2015) emphasize how boundary resources evolve through contested co-creation shaped by power asymmetries.

Recent research also emphasizes societal and regulatory implications. Aguiar and Waldfogel (2021) show that platforms like Spotify dominate suppliers and steer consumer behavior, raising antitrust concerns. Cutolo and Kenney (2021) and Lv and Schotter (2024) highlight dependency and uncertainty for platform-dependent entrepreneurs due to unilateral owner control, calling for updated regulatory frameworks. Gawer (2021a) and Jacobides et al. (2024) note that platforms, while solving coordination failures, can introduce exploitation risks and societal backlash, necessitating stronger oversight. Finally, Rahman et al. (2024) emphasize asymmetrical power through data, algorithms, and rules, advocating integrated accountability mechanisms combining bottom-up resistance and top-down regulation.

As summarized in Table 1, these studies collectively underscore the growing dominance of platform owners and the resulting concerns about fairness, prompting calls for more robust regulatory frameworks and hybrid accountability mechanisms.

**Table 1:** Overview of existing research on power dynamics in digital platform ecosystems

Study	Focus	Main findings	Regulatory perspective
Perrons (2009)	Trust and power in platform leadership	Platform leaders balance trust and coercion to maintain ecosystem control, often disguising power as collaboration and exposing complementors to risk	Not explicitly discussed
Tiwana et al. (2010)	Evolutionary dynamics of software-based platform ecosystems and their governance	Platforms evolve through a balance of platform owner control and developer autonomy, creating tension as platform owners maintain significant power	Not explicitly discussed
Eaton et al. (2015)	Boundary resources in service systems with digital technology	Boundary resources evolve through contested co-creation, often shaped by power asymmetries as platform owners control innovation	Not explicitly discussed
Aguiar and Waldfogel (2021)	Platforms’ power to influence consumer behavior and market outcomes	Platforms like Spotify dominate suppliers and steer consumer behavior through curated content, affecting market outcomes	Implied need for scrutiny of platform practices, particularly in antitrust contexts
Cutolo and Kenney (2021)	Power asymmetries in platform-dependent entrepreneurship	Platform-dependent entrepreneurs face significant dependency and uncertainty due to unilateral control by platform owners, creating power imbalances	Implied need for regulation to address power asymmetries
Gawer (2021a)	Digital platforms as emblematic organizational forms of the digital age	Platforms gain power through centrality but risk societal backlash due to potentially exploitative behavior	Strong call for regulation to address societal concerns and ensure fairness
Hurni et al. (2022)	Power paradox in platform owner-complementor partnerships	Platform power unfolds cyclically and reciprocally, with complementors actively shaping outcomes despite owner dominance	Not explicitly discussed
Lv and Schotter (2024)	Resource dependence and strategic constraints imposed by powerful platform owners	Powerful platform owners dominate digital firms by creating structural and technical dependencies that limit autonomy—a Faustian bargain	Strong call for updated antitrust laws to address dominance of powerful platform owners
Hunt et al. (2024)	Power dynamics in digital platform governance	Platform governance is shaped by coercive, manipulative, and algorithmic power, allowing owners to dominate stakeholders	Implied need for regulation to address power imbalances and ensure accountability
Jacobides et al. (2024)	Platforms and ecosystems as solutions to economic failures and their inherent faults	While platforms solve coordination failures, they introduce new functional and distributional issues as orchestrators dominate and exploit complementors	Strong call for regulation to address orchestrator abuse and ensure fairness
Rahman et al. (2024)	Platform accountability in multisided digital platforms	Platform owners hold dynamic, asymmetrical power through data, algorithms, and rules, prompting calls for integrated bottom-up resistance and top-down regulation to ensure effective accountability	Strong call for regulation and third-party oversight

Given the increasing power of platform owners in their ecosystems, concerns over power imbalances have led to increasing calls for external regulation. While self-regulation has been discussed (Cusumano et al., 2021; Jacobides & Lianos, 2021), recent literature has shown interest in exploring formal regulatory interventions aimed at correcting these power asymmetries (Gleiss et al., 2023; Heimburg & Wiesche, 2023). This is particularly relevant in light of practices such as preferential rule-setting and algorithmic opacity (Furman et al., 2019), which amplify platform owners’ power over complementors.

Regulating digital platform ecosystems is challenging, as platforms use evolving algorithms and rules to shape participation and value flows (Kokshagina et al., 2023). Their central role affects complementors, markets, and consumer choice (Baldwin & Cave, 2020), making it hard to balance innovation with limiting anti-competitive behavior (Kenney et al., 2019).

One key challenge lies in understanding the actual effects of regulation on market dynamics and power relations. While regulatory instruments such as price caps or transparency obligations aim to improve fairness (Kircher & Foerderer, 2024; Li & Wang, 2024), they may produce unintended consequences—disincentivizing innovation or even reinforcing existing power structures (Rietveld & Schilling, 2021). Moreover, platform owners may engage in strategic compliance to maintain dominance while appearing to adhere to regulatory norms (Lanamäki et al., 2025). The open texture of law allows for varying interpretations, complicating enforcement and enabling circumvention. Additionally, Simone and Laudando (2025) warn that one-size-fits-all rules risk stifling innovation by ignoring key differences in competition and rent types.

Despite these growing debates, IS research has shed little light on how the power relationships between complementors and platform owners are negotiated and transformed under regulatory scrutiny. Cutolo and Kenney (2021), for example, highlight the structural dependence of entrepreneurs on platforms but do not explore how this dependence evolves when regulation is introduced. Hurni et al. (2022) showed that power dynamics unfold as reciprocal cycles between platform owners and complementors in enterprise software ecosystems, but their findings are limited to unregulated settings and do not examine how regulations alter these dynamics. Finally, Gleiss et al. (2023) identify unequal bargaining power as a market failure in platform-based markets, particularly when platform owners exploit their powerful position, and explore possible regulatory responses, yet stop short of examining how regulatory scrutiny affects the ongoing negotiation of power within ecosystems.

In sum, while the literature has acknowledged the need for regulation in response to rising platform dominance and provided valuable insights into the consequences of regulations, it has yet to investigate how power is contested, negotiated, or reinforced within ecosystems subject to regulation. The legislative process of the DMA and the discourse around it provide a unique opportunity to study the interactions of complementors and platform owners.

## The EU’s Digital Markets Act (DMA)

**Fig. 1:**
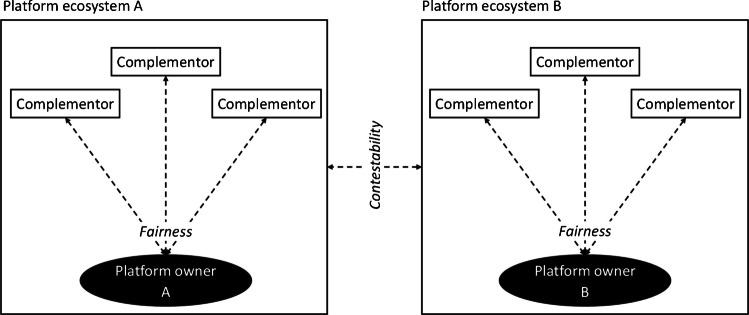
Contestability and fairness in digital platform ecosystems

The EU’s Digital Markets Act (DMA) represents a landmark regulatory intervention aimed at strengthening the EU’s internal market and is designed to promote fairness within and contestability across digital markets (Fig. 1) (EU, 2022). Unlike traditional antitrust frameworks, which react to anti-competitive behavior after it has occurred, the DMA uses an ex ante approach, imposing clear obligations to prevent abuses of market power. By proactively targeting dominant platform owners designated as “gatekeepers,” the DMA reflects a broader shift toward addressing power asymmetries that impact fair competition and innovation.

Gatekeepers are platform owners with a substantial market impact, offering essential core platform services that serve as pivotal gateways for businesses to reach customers. The DMA defines them based on financial and user thresholds, including an annual EU turnover exceeding €7.5 billion or a market capitalization of at least €75 billion, as well as over 45 million monthly active end users and 10,000 active business users in the EU. Designated in September 2023, gatekeepers, namely Alphabet, Amazon, Apple, ByteDance, Meta, and Microsoft^1^ had until March 2024 to comply with provisions including bans on self-preferencing practices, ensuring fair treatment of complementors, and enhancing transparency for consumers. The provisions affect various digital services such as advertising, search, and social networks. For instance, Apple and Google have been designated gatekeepers for their online intermediation service, the Apple App Store and Google Play Store.

The development of the DMA unfolded between 2020 and 2024 through an intense phase of policy design, negotiation, and lobbying. Following the European Commission’s initial proposal in December 2020, the DMA was debated extensively within EU institutions and among stakeholders. Phases of negotiation, designations, and application occurred until the compliance obligations were officially enforced in March 2024 (European Commission, 2020).

During this developmental phase, major technology firms, including Alphabet, Apple, Meta, Amazon, and Microsoft, engaged in unprecedented lobbying efforts aimed at shaping the regulation’s scope and obligations. Reports highlight how lobbying increasingly occurred through digital channels, with Big Tech companies mobilizing think tanks, trade associations, and even government allies to influence the narrative around innovation and competition (Corporate Europe Observatory, 2020; Scott & Kayali, 2020). Public consultations, expert workshops, and roundtables organized by the European Commission between 2022 and 2023 invited feedback from industry, consumer organizations, and civil society on issues such as self-preferencing bans, interoperability, and data-sharing obligations. This period of negotiation and dialogue was crucial in defining the final contours of the DMA.

The DMA can be seen as a tool to reshape power dynamics within digital platform ecosystems by imposing constraints on dominant players and promoting a more equitable value distribution. It contributes not only to the enforcement of competition rules but also, more broadly, to the governance of digital ecosystems (Gleiss et al., 2023; Heimburg & Wiesche, 2023). However, the open texture of law (Lanamäki et al., 2025) implies that regulatory language contains inherent ambiguities, allowing powerful gatekeepers to strategically interpret or comply with new rules, potentially limiting the effectiveness of regulation.

Emerging empirical work suggests that the effects of the DMA are still unfolding and not yet fully understood. Early evidence from companies like Amazon indicates strategic behavioral shifts rather than structural change, emphasizing the need for more research into how regulatory pressure translates into altered power relations (Aguiar & Waldfogel, 2021). As Simone and Laudando (2025) note, the DMA’s effectiveness hinges on recognizing the varied sources of gatekeeper power and types of economic rents they extract. Without this nuance, uniform obligations risk suppressing not just anti-competitive practices but also legitimate value-creating activities.

By targeting gatekeepers, the DMA aims to rebalance power in ecosystems, highlighting the need for theory development on how such regulation affects platform-complementor dynamics. This study focuses on how the DMA, in its regulatory development phase, shapes power dynamics and the potential implications for fairness and contestability within and across ecosystems, particularly in light of the extractive practices that may persist despite regulatory efforts (Rahman et al., 2024).

## Method

We conducted a revelatory case study of the DMA utilizing an embedded, single-case design study (Yin, 2014), delving into two types of digital platform ecosystems: app stores (Google and Apple) and search platforms (Google). We investigated how power is negotiated during the regulatory development phase (regulatory scrutiny). This approach allowed us to examine the complexity of digital ecosystems and how actors engage in power contestation during policy formation.

By studying the discourse surrounding the DMA before its enforcement and focusing on the period during which regulatory ideas were formed, negotiated, and contested, we examine how platform owners and complementors actively sought to shape the regulation’s framing and thus influence their position in the ecosystem.

We define the DMA discourses as the major topics in public opinion on the (proposed) regulation. As the constituent parts of the discourses, we regard any publicly available position from stakeholders aimed at shaping the regulation’s design. By analyzing the discourse on the proposed and adopted regulation, we have a revelatory case for understanding actors’ positions, interactions, and dependencies in platform ecosystems (Yin, 2014). In doing so, we can better understand actors in broader platform ecosystems, their positions toward openness, boundary resources, value creation and capture, and how power is contested throughout regulatory formation processes.

We focus explicitly on the power struggles and strategic behaviors leading up to the finalization of the DMA, not its post-enforcement effects. Focusing on app stores and search platforms, we analyze the data using grounded theory methodology, identifying key actors, arguments, and relational dynamics across digital platform ecosystems. We chose the grounded theory methodology to explore the DMA because its unique regulatory framework and emphasis on fairness and contestability represent an emergent phenomenon within platform regulation. This approach allows for inductive theory-building grounded in the qualitative data from the DMA’s legislative process.

### Data collection

**Fig. 2:**
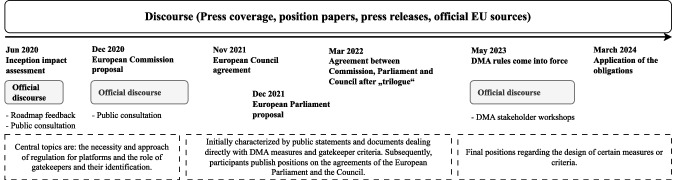
Timeline and key events in the DMA discourses

We collected data from June 2020 (start of the official legislative work) to March 2024 (application of the obligations), covering the full duration of the DMA’s development. Sources include official European Commission forums and consultations, as well as major newspapers and industry media. Figure 2 highlights the timeline and key events in the DMA discourse.

We purposefully decided to focus on the DMA as a whole for data collection, but concentrated our analysis on the app store and search platform ecosystems. Their significant influence on the digital market landscape made them central to our investigation, providing a focused lens through which to understand the broader implications of the DMA regulations.

We identified the contributions to discourses in various channels (Table 2). The discourse participants contributed to the official forums and public consultations for policy discourses provided by the European Commission. Also, the participants contributed to unofficial channels of the discourse. The majority of the unofficial channels are newspaper articles we identified by searching with the query “Digital Markets Act” on Dow Jones Factiva, Nexis Uni, Politico, and Euractiv.^2^ First, this allowed us to discover statements of participants that the newspapers reproduced and interviews with participants. Second, including the press coverage also allowed us to analyze the contributions of political participants in the discourses. Third, we retrieved further unofficial contributions, such as position papers, press releases, and other statements, through a backward search of the identified newspaper articles. Fourth, using multiple data sources ensured the corroboration of the evidence.

### Data analysis

After reviewing the available data and considering the objective of studying power dynamics, our analysis concentrated on documents specifically addressing app store and search platform ecosystems. For our data analysis, we primarily draw on the Straussian approach to grounded theory methodology, utilizing open, axial, and selective coding, along with memoing to enhance our understanding of the data (Seidel & Urquhart, 2014; Strauss & Corbin, 1990; Wiesche et al., 2017). We conducted an additional round of coding, through Anselm Strauss’s concept of a coding paradigm, to further develop categories and identify central phenomena (Bryant & Charmaz, 2007; Strauss & Corbin, 1990).

We started the data analysis by obtaining a foundational understanding of the discourses on the DMA. We began by coding newspaper articles and progressed to participants’ contributions with decreasing heterogeneity until we coded the contributions of GAMMA^3^ companies, which are the main target of the DMA. Out of the initial 1723 documents collected, we selected 417 documents and three videos (equivalent to 23 hours of official European Commission workshops on the DMA) for in-depth analysis and coding. These were chosen based on their relevance to the Apple App Store, Google Play, and Google Search ecosystems. We excluded documents because they were either unrelated to the platforms studied, repetitive in content, or mentioned the DMA only in passing without relevant analytical value. While all collected documents informed our overall understanding, the 417 selected items formed the core dataset from which we developed our codes and conceptual model. Searching for relevant discourses, narratives, positions, and actors, we totaled 1464 codes for 5070 quotations. In the results section, we primarily use quotes from the DMA workshop video transcripts (Table 2) to highlight each actor’s direct input, thus avoiding information that has already been processed (i.e., press articles). All references in the results section—both direct and indirect quotes—are linked to document numbers (e.g., D107), which are listed in the Appendix along with their sources.

**Table 2:** Sources of contributions to DMA discourses

Source		Details	Time	# of documents	# of selected documents
**Official**	DSA package^a^	Roadmap feedback on inception impact assessment	Jun 2020	30	30
		Public consultation	Jun–Sep 2020	22	22
	Single Market^b^	Roadmap feedback on inception impact assessment	Jun 2020	16	16
		Public consultation	Jun-Sep 2020	16	16
	DMA Proposal for a regulation	Feedback on European Commission adoption	Dec 2020– May 2021	20	20
	DMA workshop videos	Applying the DMA’s ban on self-preferencing: how to do it in practice? (W1)	Dec 2022	1	1
		The DMA and Apple App Store-related provisions (W2)	Mar 2023	1	1
		The DMA and data-related obligations (W3)	May 2023	1	1
**Unofficial**	Press coverage (particularly in specialized press)	Politico	Jun 2020– March 2024	617	114
		Reuters		155	35
		Financial times (FT)		98	28
		Euractiv		97	34
		Others		335	30
	Position papers, press releases, and other statements			314	69
			**Total**	**1723**	**417**

Notes: ^a^The Digital Services Act (DSA) regulates online platforms to ensure user safety, prevent harmful activities, and promote fundamental rights. The DSA was adopted along with the DMA, creating a single set of rules applicable across the EU. ^b^The Single Market’s new complementary tool strengthens competition enforcement across the member states of the EU by addressing structural issues, ensuring fair, competitive, and innovation-driven markets

Organized by the European Commission, these DMA-related workshops enabled interested stakeholders to express their views on specific issues related to the legislation’s framework and discuss effective compliance by gatekeepers. The first workshop focused on banning self-preferencing (W1), and included panelists such as BEUC, EU Travel Tech, Google, Yelp, Expedia, and Jobindex. The second workshop addressed Apple App Store provisions (W2) and featured Spotify, Apple, BEUC, Match Group, and the Developers Alliance. The third workshop related to the DMA and data-related obligations (W3), with participants like Meta, Google, Zalando, Amazon, EDRi, and CNIL.

To analyze the dynamics within the two types of platform ecosystems—app store and search platforms—we applied open coding using Atlas.ti (Muhr, 2008) to identify discourses, narratives, positions, and actors. Through this process, we identified three primary actors involved—the platform owner, the complementor, and the regulator—and various discourses as they interacted with the European Commission and the regulatory framework. Through line-by-line coding, we captured both self-perceptions and perceptions of others, revealing arguments claimed or defended by the actors. For instance, what a complementor referred to as “platform owner avoiding gatekeeper status” was portrayed by the platform owner as “ensuring transparency and fairness.” From the data, we identified position-making behaviors among the actors who either claimed or defended their arguments. As the open codes reflected the thoughts and positions of each actor in relation to one another, our second-order themes emerged as the arguments articulated by each actor within the ecosystem. Through axial coding, we henceforth organized concepts into categories and explored their interrelationships. Through this process, we identified topics such as “intent to comply” by platform owners and “regulatory non-compliance” by complementors.

Selective coding further refined these categories, revealing signs of power dynamics. We identified relationships between the categories that illustrated different types of “Power contestation,” such as accusations directed at platform owners and demands made to regulators by complementors, as well as defensive arguments from platform owners towards complementors and warnings or intimidation directed at regulators. To further integrate and abstract our findings, we engaged in Anselm Strauss’s concept of a “coding paradigm” (Bryant & Charmaz, 2007; Strauss & Corbin, 1990), iteratively examining the relationships between our finalized categories. This enabled us to conceptualize higher-order process categories—"Weak power assertion,” “Power position protection,” “Power negotiation,” and “Power shift advocacy”. These represent distinct but interrelated forms of power contestation during the rule-development phase. We identify “Power contestation” as the emerging core category, reflecting the tensions and struggles between actors within the DMA context. It is important to note that contestation, as an expression of power dynamics, could manifest differently in other fields and cases depending on the specific actors and the nature of the ecosystem.

In total, we identified 31 arguments (16 in app store platform ecosystems and 15 in the search platform ecosystem) and eight aggregated dimensions. Using “Power contestation” as the core category of our coding procedures, we develop a conceptual framework integrating the findings across the two types of ecosystems, which we explain through four propositions (Cornelissen, 2016). The complete overview of our coding scheme following Gioia et al. (2013) can be found in the Appendix. We used memoing to make notes, summarize the major positions in the discourse, and write interpretations of how participants engaged in the discourses. Although we began the coding process with little preconceived notions, we oriented our analysis towards power dynamics. As the coding process advanced, more apparent arguments emerged from the platform ecosystem actors regarding the regulation, particularly as they took positions throughout the regulatory process, from the initial draft of the DMA to its implementation. By aggregating participants from a single organization before analyzing their discourses, narratives, and positions, we could examine these elements at an institutional level.

We now analyze power dynamics in the app store and search platform ecosystems. As part of a cross-case analysis, we aggregate the findings into a cohesive framework that highlights elements of power contestation, which we explain through four propositions (Cornelissen, 2016). This allows for a deeper understanding of how power is contested and negotiated across digital platform ecosystems during regulatory formation. We first outline the results of the app store platform ecosystems, then the search platform ecosystem. We then describe our unified framework of power contestation in digital platform ecosystems under regulatory scrutiny.

## Results

We show how the DMA shapes power dynamics through power contestation behaviors within platform ecosystems. First, in the app store ecosystems, then in the search ecosystem, we identify key actors and analyze their interactions through various types of argumentations. The regulator is positioned as the recipient of competing claims and pressures from both sides: complementors raise accusations, demands, and concerns regarding fairness and regulation, while platform owners offer defensive arguments and assert a commitment to compliance. These contestations reflect an instantiation of power dynamics within the context of the DMA, which aims to ensure fair competition by imposing obligations on gatekeepers.

### Power contestation in app store platform ecosystems

An app store or software application store, defined by the EU as an online intermediation service focused on software applications, is one that *allows business users to offer goods or services to consumers, with a view to facilitating the initiating of direct transactions between those business users and consumers, irrespective of where those transactions are ultimately concluded* (EU, 2019). The service provider, i.e., the platform owner, in the case of the app store platforms, includes both Apple and Google, both of which host a plethora of applications on their respective app stores. Their service is based on contractual relationships with business users, who in this case include third-party app developers—i.e., complementors—who benefit from the app stores to present and distribute their applications.

We interpret app store platform ecosystems as hybrid platforms because they typically comprise innovation and transaction platforms. The innovation platform is a technological foundation for complementors to develop third-party applications (Android and iOS in the case of Google and Apple). The transaction platform is the marketplace that distributes the application to end users (Google Play Store and the Apple App Store). While the DMA refers to app store platform ecosystems as online intermediation services and thus as a transaction platform, the underlying innovation platform is vital to Google’s and Apple’s market power as providers of ecosystems for mobile apps.

Beyond Google and Apple as the two leading actors, the regulator plays an increasingly important role, setting out rules that can significantly alter the dynamics of the ecosystem. With a high level of legal power, many actors participate in the ecosystem to influence the regulator. In addition, associations, whether representatives of complementor organizations or others, are another type of actor in the ecosystems. By representing several smaller organizations, they could hold their position against giants such as Apple and Google.

We also differentiate two groups of complementors that differ in size and power (Asadullah et al., 2023). On the one hand, large and well-established companies such as Spotify or Match Group occupy a secure and distinct place in the market and are looking to strengthen their position by ensuring that regulations benefit them. On the other hand, small and medium-sized enterprises (SMEs), such as small app developers who rely heavily on platforms, adopt a slightly different stance. Fearing the regulation’s negative impact on them, they recognize the need for platform owners to exist and support them.

The DMA regulation, requiring platform owners to ensure fairness within ecosystems, should ultimately offer app developers a fairer environment in which to offer their services and more choice for end users, better services, and fairer prices.

**Fig. 3:**
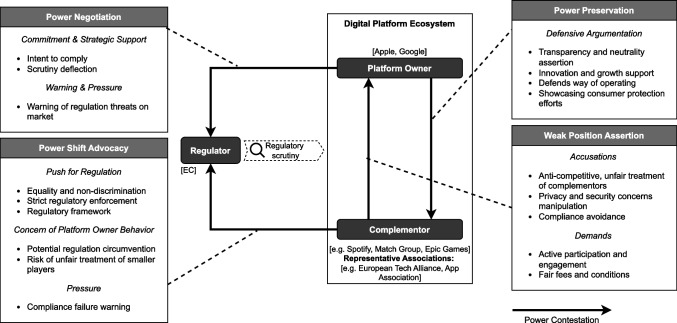
Power contestation in app store platform ecosystems

Under regulatory scrutiny, we identify distinct relationships between each ecosystem actor. In parallel to the direct relation between the platform owner and complementor, additional relations, spurred by a game of influence, manifest between the platform owner and the regulator and between the complementors and the regulator (Fig. 3).

#### Weak position assertion

Within digital platform ecosystems under regulatory scrutiny, complementors voice accusations and demands towards the platform owner, blaming them of unfair, anti-competitive, and restrictive practices such as content censorship and control, unfair financial practices, and user experience and interface manipulation (D591, D1045). France Digitale condemned the platform owners, saying: *Because of their gatekeeper power, Apple and Google are able to impose unfair and discriminatory terms and conditions on app developers. If you are an app developer and you want to distribute your app to smartphone users, Apple and Google will impose several unacceptable constraints* (W2, France Digitale). In addition, complementors accuse platform owners of using security and privacy concerns (D691) as a false pretense to assert more decisional power over complementors and avoid regulations (W2, Open Web Advocacy, Schibsted). When speaking of actions taken by Google or Apple to protect their app store platform ecosystems, the European Games Developer Federation warned that their actions *should strictly be limited to essential legal, compliance, trust, privacy and security and safety concerns* and that *these measures should not be used as an excuse for censorship* (W2, European Games Developer Federation). By accusing platform owners of discriminatory practices, complementors showcase their weaker position in their relationship with the platform owner (D543). Through a host of examples and demonstrations, complementors assert their disadvantageous position in the ecosystem: Apple takes a cut of as much as 30% of revenue generated by apps distributed through its digital store and imposes rules on content and other practices (D672).

#### Power shift advocacy

In parallel, complementors advocate for a shift or rebalance of power in the ecosystem by conducting an influential plea to the regulator (D787, D625). By pushing for more or stricter regulations, they aim to toughen the stakes for platform owners. Our results show a push for more equality and non-discrimination. The App Association—representing small app developers—urges to prevent large companies from leveraging their size unfairly: *It will be essential to allow genuine participation from all stakeholders during this implementation phase to ensure that the DMA is efficient and does not favor the largest app makers at the expense of the smallest ones* (D1041).

Strict enforcement has been described as crucial to the success of the regulation, arguing that all efforts will depend on ensuring strict and timely application of the rules to platform owners (D16). Spotify (a complementor) encouraged the regulations, arguing that successful compliance will lead to leveling the playing field for app developers on platforms, and urged for complete and strictly enforced rules (D1137, D1138), adding that *all of the applicable DMA provisions will be important to truly addressing the gatekeeper power of these platforms and achieving the goals of the DMA* (W2, Spotify).

Complementors expressed significant concern about the eventuality of platform owners circumventing regulations (D1139, D625), applying pressure to highlight the risks of gatekeeper non-compliance and the potential legal consequences. They asked the regulator to anticipate certain circumvention attitudes. Spotify, aware of the risks, suggested: *All of the provisions of the DMA applicable to app stores and mobile operating devices will necessarily have to work together to cut off avenues for circumvention, whether that takes the form of impeding alternative means of distributing and downloading apps, imposing discriminatory or unreasonable terms on developers, or throwing sand in the gears on interoperability* (W2, Spotify).

Beyond the plea for more equitable power in the ecosystem, SMEs feared that the DMA would negatively impact them, thus putting them at a disadvantage compared to their larger competitors. *The DMA risks generating unintended consequences that could indirectly affect SMEs’ business models, growth ambitions, or exit strategies. Gatekeepers may recoup their losses in ways that harm SMEs, such as reducing their investments in platform infrastructure and tools that benefit SMEs, and thereby increase the cost of entry for smaller actors* (D678). In addition, there was a recognition that platform owners were essential to protect against piracy and malware and, therefore, the survival of smaller app developers with fewer resources than their larger competitors (D906, D678).

#### Power preservation

Within their dominating positions, the platform owners sought to keep their advantageous position in the ecosystem. By defending its ways of operating and claiming to support innovation and growth within its ecosystem, Google reinforced the notion that its platform offers valuable opportunities for developers to grow and succeed (D746). A spokesperson for Google, when demonstrating the value of their platform for complementors, argued: *You have two million developers in constant phases of experimentation and entering with new features, with new applications expanding, and you need to have a model that’s designed to accommodate that kind of activity* (W2, Google), with Apple adding that they have d*elivered choice, competition and innovation for European consumers, helped developers start and grow new businesses* (D679).

For many accusations, platform owners argued in defense of how they operate (D1087). For instance, regarding accusations of unfair fee policies, Google argued that its fee policy approach aligns with market practices: *It’s not just about the level of the fee; of course, it’s about how you structure the fee. It’s about how you set the access conditions more generally. […] It will come as no surprise to you that we look at the market and see that the model that we have adopted is not unique* (W2, Google), adding that *some rules, including data portability, fee transparency, privacy and choice of services, should apply to all companies and not only the largest ones* (D107).

Ensuring the privacy and security of Europeans is a key argument, with platform integrity highlighted as crucial to unique features and consumer protection efforts (D691). In an effort to strategically justify its platform control, Apple argues: *At a time when threat vectors related to data theft are multiplying exponentially, we operate with an unwavering commitment to Europeans’ privacy and data security. […] We are dedicated to providing our customers with high-quality, reliable, and easy-to-use devices and features. […] How to maintain Apple’s commitment to our fundamental principles while at the same time ensuring that our products and services comply with the DMA? Our customers’ trust means the world to us* (W2, Apple).

#### Power negotiation

While platform owners defended their operating mode and maintained that a balance of power and fairness exists concerning complementors, they also negotiated their power with the regulator (D746). Under the regulatory threat, the platform owners strived to create a collaborative relationship with the regulator, demonstrating their intention to comply and engage with the regulator to discuss key aspects of regulation.

In their negotiation efforts, platform owners warned of regulatory threats to the market, emphasizing threats to privacy and security, such as sideloading,^4^ which Google has warned would damage privacy and security (D672, D1059). Gatekeepers have made use of strategic support through implicit scrutiny deflection, emphasizing their own compliance while redirecting regulatory attention toward rivals. Apple, for instance, underscored the privacy and security risks of sideloading on Android (Google) to argue against loosening app store restrictions (D672).

Further warnings about the potential adverse effects of the regulation on the economy were expressed, as well as a caution regarding consumer mistrust potentially harming developers and the app store ecosystem. Apple expressed: *We are concerned that a loss of consumer trust will result in an [Apple] App Store ecosystem that will be less dynamic, diverse, and rewarding for developers* (W2, Apple).

### Power contestation in search platform ecosystems

The EU defines an online search engine as *a digital service that allows users to input queries in order to perform searches […] on the basis of a query on any subject […] and returns results in any format in which information related to the requested content can be found*. In the search platform ecosystem, we find the sole designated gatekeeper, Google, in the position of platform owner. Complementors, or third parties, offer their services via Google’s search platform. Thus, we interpret search platform ecosystems as transaction platforms in which complementors provide services enhancing basic search results (e.g., on hotels, flights, and e-commerce offerings), which end users draw on as part of their online searches.

**Fig. 4:**
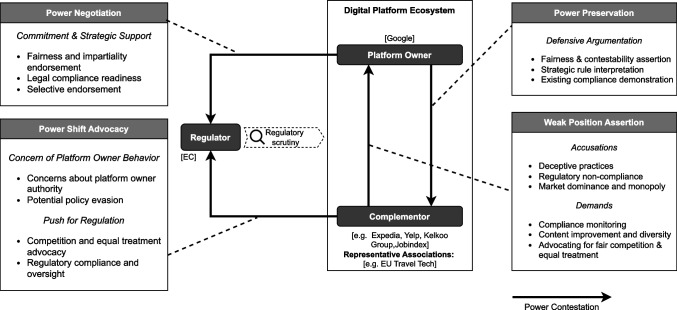
Power contestation in search platform ecosystems

Figure 4 illustrates the ecosystem with selected complementors who actively participated in the regulatory discussions. Google had started to offer additional services on its search platform, such as hotel comparisons (Google Hotels), flight comparisons (Google Flights), and e-commerce comparisons (Google Shopping). These services had previously only been offered by complementors; thus, these complementors were now increasingly concerned with fairness within the ecosystem. The regulations in force should enable non-Google services to gain visibility. Jobindex, for example, a job portal, had come into direct competition with Google’s job service and had seen Google favor its own service in the search results through preferential placement and results configuration. On the shopping side, companies such as Kelkoo or Heureka Group, which offer comparison services, had to go head-to-head with Google’s Comparison-Shopping Service (CSS).

The relationships between the various actors in the search platform ecosystem were similar to those in the app store platform ecosystem, with differences in the types of arguments put forth.

#### Weak position assertion

The complementors voiced multiple accusations against the platform owner. Foremost among these were deceptive practices, such as algorithmic biases and manipulations, which created disparities in treatment between Google’s own services and similar competing services (D837). Yelp states: *Instead of being gateways that facilitate access, large dominant platforms use their privileged position to increase their own market power* (D1011). EU Travel Tech had shown frustration as to how Google Flights dominated the search result, leaving competing flight comparison services little visibility: *We believe that this Google unit embedded in the SERP (search engine results page) is getting a differentiated and a more favorable treatment in ranking* (W1, EU Travel Tech)*.* The “bait and switch model” that led users to Google’s own online intermediation service rather than providing them with direct answers or options for their query had been widely acknowledged by complementors (D99).

Complementors further claimed that the design of the search results architecture, which favored Google’s services, using carousels, for instance, could potentially confuse users and manipulate product selection (D808). Expedia demonstrated that Google’s own services in the results, as compared to the others, had more *richness and attractiveness of the design* (W1, Expedia).

These practices have led complementors to denounce Google’s monopolistic position on its own search platform (D662). The browser DuckDuckGo clarified: *We concur that it is critical to dilute the power of online gatekeepers, for the good of society, innovation, and competition* (D722). By favoring proprietary services and controlling traffic to merchants, they argued that Google’s dominant position primarily increases its revenues while raising costs for stakeholders (W1). A spokesperson for Heureka Group clarified: *At this moment, Google has a dominant, a super dominant position on the market. A monopolistic position is harming the market* (W1, Heureka Group).

The solutions were clear to complementors, as demands were raised: search results content should be improved and diversified, allowing organic results to trump the favoritism of proprietary services (D837, D1011). A redesign would also enable fairer competition and an improved user experience.

#### Power shift advocacy

In parallel, complementors pushed the regulator to enforce fair competition and equal treatment in the ecosystem, thereby pressuring platform owners to comply with their requirements through legal obligations (D1118). Kelkoo, like its peers, pleaded for conventional competition: *Competition is on product price, availability, delivery, and brand. This is competition. This is what we used to have, and this is what the DMA should deliver* (W1).

Aware of possible circumvention strategies the platform owner may use, complementors warned that such schemes should be identified and tackled by applying strict regulations. The European Magazine Media Association (EMMA) and European Newspaper Publishers Association (ENPA)—news associations—warned that gatekeepers *can easily circumvent [provisions]…[which] would benefit the gatekeeper while negatively affecting fair competition* (D676). In addition, complementors voiced concerns over the platform owners’ use of authority, fearing that it gives them too much freedom to decide on their practices (D803). Concerning ad auctions, for example, Kelkoo asked the question: if it is not the market that decides, but Google, *can an auction be fair, reasonable, and non-discriminatory?* (W1).

#### Power preservation

The platform owners used various arguments to defend themselves against the accusations, above all, by asserting fairness on the search platform and their commitment to providing users with diversified search results and formats (D746). Through the fine dissection of the regulation, they could demonstrate their existing compliance or ensure it for the future. For example, when accused of preferential treatment, Google turned to legal manuscripts, highlighting the difficulty of defining such a term and, therefore, being accused of such treatment. *There’s a clear description of what the core platform can do as an online search engine. We can show results across all types of content. We can show results in any format. Those results can be free and paid, and they could be grouped or organized in ways that are useful to our users. What then is the thing that’s separate from what I have just described that could potentially be favored?* (W1, Google).

#### Power negotiation

Meanwhile, they maintained a collaborative relationship with the regulator by committing to regulatory compliance and making themselves available for discussions (D746). Google clarified its position towards the regulator: *It is clear that the DMA is now law. We have to obey the text, and we have to be ready to make changes. And we are. There will be changes to the SERP as a consequence of the Digital Markets Act*, adding *Yes, we have to expect an ongoing dialogue with the Commission across all of our obligations* (W1). Under the threat of strict rules and legal consequences, platform owners adopted a negotiating stance towards the regulator compared to a defensive one when dealing with complementors (D925). Platform owners engaged in strategic support for regulation, selectively endorsing regulatory principles that aligned with their interests. For instance, Google advocated for a *baseline of high-level principles that could be applied across different types of platforms […] complemented by platform-specific guidance* (D107). This selective endorsement reflects an attempt to steer regulatory design in a direction that legitimizes compliance while minimizing disruption to their business practices.

In the next section, we present the observations arising from a cross-case analysis carried out in the ecosystems studied. This approach enabled us to identify recurring patterns and dynamics, which were synthesized in a conceptual model and used as a basis for propositions.

## A conceptual framework of power contestation in digital platform ecosystems

**Fig. 5:**
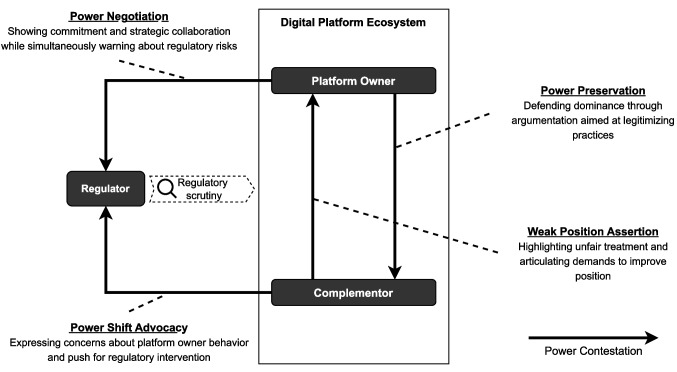
Framework of power contestation (in digital platform ecosystems under regulatory scrutiny)

We conduct a cross-case analysis to develop a conceptual framework (Jabareen, 2009; Miles et al., 2014) that captures emergent patterns of power contestation among platform owners, complementors, and regulators within digital platform ecosystems under regulatory scrutiny (Fig. 5). This approach allows us to compare and contrast the relationships across the different ecosystems (app store and search platforms), providing insights into how power is negotiated and contested in the context of regulatory frameworks such as the DMA.

Across the app store and search platform ecosystems, we identified a recurrent pattern of power contestation between the platform owners and complementors, with the regulator positioned as the recipient of competing claims and pressures from both sides. In the direct interaction between the complementors and the platform owner, complementors assert their weak position while platform owners strive to preserve their position of power. In both ecosystems, complementors voiced accusations towards platform owners, such as unfair treatment of complementors in the app store platform ecosystems and deceptive practices in search platform ecosystems (Gawer, 2021a). Platform owners, in response, tried to preserve their power, for example, by claiming that their overarching goal was to foster innovation and growth of the app store platform ecosystems. Platform owners also negotiate their power through interaction with the regulator by actively engaging in the discourse and offering strategic support for certain regulatory principles while warning of the potential negative consequences of overregulation for the market. At the same time, complementors advocate for a power shift by leveraging their weaker position to push for stricter enforcement and broader regulatory constraints on platform owners, aiming to rebalance power within the ecosystem.

Power dynamics are not static; they are continually contested, negotiated, and redefined through mechanisms of contestation that are both apparent and subtle. The relationship between complementors, who claim a weak position of power, and platform owners, who defend their dominant position, often manifests itself in a struggle for control.

Based on an analysis of the dynamics between platform owners, complementors, and regulators within digital platform ecosystems, we identify four forms of interaction that characterize how power is contested when these ecosystems come under regulatory scrutiny: platform owners’ power negotiation with regulators, complementors’ advocacy for a power shift, platform owners’ efforts to preserve their power, and complementors’ assertions of weak power.

### Platform owners’ power negotiation

In early regulatory debates, platform owners engage in strategic collaboration with regulators, positioning themselves as innovation enablers and critical market players, in order to influence how regulations are shaped. They interpret and define regulatory requirements according to their interests, enabling them to retain control. These interactions combine engagement and strategic collaboration with warnings against regulatory excess and market damage.

Platform owners use defense strategies that often involve maintaining a dominant discourse that justifies their actions. In the case of the DMA, this can include attempts to resist regulatory oversight or manipulate the interpretation of regulations to protect their interests. This resistance to external regulation can be seen as a form of power, with platform owners using their influence to shape perceptions of the regulator’s role and legitimacy. However, as Fleming and Spicer (2014) suggest, this discursive power has limits. When regulators detect instances of non-compliance or system manipulation, they can trigger a shift in power dynamics, leading to stricter regulations and increased oversight. In sum, we draw the following proposition:

Proposition 1: In digital platform ecosystems under regulatory scrutiny, platform owners engage in power negotiation with the regulator—through strategic collaboration, commitment to select regulatory goals, and warning of potential harms—to preserve their power in the ecosystem.

By presenting themselves as essential to economic growth and market efficiency, platform owners seek to create a narrative that reduces the legitimacy of regulation. As a result, these tactics contribute to gradually weakening the intended impact of the regulation.

### Complementors’ advocacy for power shift

Complementors that participate in the regulatory discourse, and who often occupy a relatively weaker position within platform ecosystems, actively seek to alter the power dynamics in their favor. Their efforts to rebalance power within the ecosystem are rooted in the need to reduce the dominance of large platforms and gain market power. While not all complementors engage in such actions, those with sufficient resources or strategic interest engage in strategic advocacy to influence regulators, pushing for stricter regulations and legal constraints on powerful platform owners. However, their advocacy is not always without resistance. Platform owners, aware of these efforts, may try to undermine or delegitimize these actions, presenting them as attempts to disrupt innovation. From this, we derive the following proposition:

Proposition 2: In digital platform ecosystems under regulatory scrutiny, complementors advocate for a power shift with the regulator to limit the power of platform owners—by expressing concerns and pushing for regulatory intervention.

By highlighting market distortions, complementors advocate for regulatory intervention to level the playing field. Platform owners challenge these narratives by presenting themselves as innovators and job creators. By deflecting responsibility, the actors fall into intangible tensions, paving the way for the need for regulation. Complementors capitalize on this tension to encourage regulatory intervention that should reduce the influence of platform owners.

### Complementor’s weak position assertion

Complementors draw attention to their weaker position within platform ecosystems and attempt to assert themselves by openly criticizing platform owners’ practices and calling for regulatory efforts. These assertions are often formulated as accusations of unfair, anti-competitive, and discriminatory behavior on the part of platform owners. By portraying platform owners as powerful gatekeepers who exploit their position, complementors attempt to draw attention to the asymmetries that define their relationship. By demanding greater transparency, fairer access, and design neutrality, complementors seek to frame their vulnerability as a legitimate reason to strengthen law enforcement. Their efforts to expose imbalances and harm serve both to prove their marginality and to encourage regulatory action. These insights lead us to the following proposition:

Proposition 3: In digital platform ecosystems under regulatory scrutiny, complementors assert their weak position by voicing accusations and issuing demands that highlight platform owners’ dominance and call for regulatory intervention to rebalance ecosystem dynamics.

### Platform owners’ power preservation

In turn, platform owners articulate justificatory defenses to preserve their dominant position and contain threats to their control, often by strategically framing regulatory challenges in ways that protect their interests (Hunt et al., 2024). Their strategies include defending existing practices, asserting a non-discriminatory and fair role, and emphasizing their contributions to innovation, consumer protection, complementor value creation, and overall ecosystem growth. By promoting a discourse that emphasizes their public and economic value, platform owners seek to legitimize their structural power (Jacobides et al., 2024). They also resist the demand for regulation by positioning themselves as responsible players already in compliance or capable of self-regulation. These arguments enable them to contest the need for intrusive surveillance and maintain the status quo. In doing so, platform owners transform defensive responses into strategic maneuvers aimed at reinforcing their authority. We thus form the following proposition:

Proposition 4: In digital platform ecosystems under regulatory scrutiny, platform owners preserve their power by defending their practices and promoting narratives of innovation, compliance, and market value, thereby resisting regulatory impositions.

The dynamics within platform ecosystems are marked by entrenched positions, with complementors and platform owners locked in a continuous cycle of contestation reflecting the reciprocal and evolving nature of power relations identified by Hurni et al. (2022). This contestation creates persistent tensions, with both sides holding firm to their positions. Camped in their positions, the tension between platform owners and complementors leads to a regulatory impasse, where efforts to resolve the power imbalance are constantly undermined by the persistent contestation between the two players, limiting the real impact of regulatory interventions.

## Discussion

As the European Commission started the legislative process of the DMA, both complementors and platform owners saw this as an opportunity to voice their position to the Commission. Building on prior research on power dynamics within digital platform ecosystems (Hunt et al., 2024; Hurni et al., 2022; Lv & Schotter, 2024; Tiwana et al., 2010), we find that both platform owners and complementors engage strategically to influence regulations. In app store and search ecosystems, complementors advocated for a power shift in a bid to strengthen their position of power in the ecosystem. Platform owners negotiated their power by interacting with the regulator, intending to maintain their powerful position in the ecosystem. This is consistent with Perrons (2009), who finds that platform leaders balance coercion and trust to retain control under the guise of collaboration, and Hunt et al. (2024), who describe how platform owners use coercive, manipulative, and algorithmic forms of power to legitimize their dominance. Across both ecosystems, we have seen that power contestation involves not only the platform owner and complementors individually but also the interest groups and associations formed by these actors. For example, complementors in the app store platform ecosystems aim to strengthen their position and have a stronger voice by forming associations like the European Games Developer Federation or EU Travel Tech. This pattern resonates with Hurni et al. (2022), highlighting that complementors can shape outcomes through reciprocal power cycles, despite owner dominance. While the overall process of power contestation was similar in the app store and search platform ecosystems, we identified nuanced differences in how the process unfolded.

First, in app store ecosystems, Apple and Google dominate, while in search, Google is the sole gatekeeper. As a result, some app store complementors, especially larger ones like Spotify, retain some power due to multi-homing (Cenamor, 2021). In contrast, search complementors depend entirely on Google, making them more vocal in advocating for regulatory change, particularly over value capture and self-preferencing. This suggests that “weak position assertion” applies more to smaller or dependent complementors, while larger players engage in more strategic contestation.

Second, the ecosystems differ conceptually: the app store is a hybrid platform (Cusumano et al., 2019), while search is a transaction platform. App store complementors focused on distribution and value capture constraints, while search platform complementors criticized self-preferencing (Furman et al., 2019). Platform owners also used different defenses: Apple and Google emphasized app quality and security, while Google framed search results as optimizing user experience. Lastly, the complementor group dynamics also diverged. In the app store platforms, larger complementors had more leverage, while smaller ones were more dependent and often formed associations. In search, concerns centered on competition with Google’s own services, rather than other complementors.

These differences highlight which aspects of our framework are more robust across cases, and which require more caution. In particular, the concept of power contestation and the key roles played by platform owners, complementors, and regulators are consistently observed in both ecosystems, suggesting a strong empirical foundation for these elements. However, aspects such as complementor positions, the forms of advocacy, and the defensive strategies of platform owners vary depending on the markets, their structure, and actors. These variations call for careful consideration when generalizing these dynamics to other settings.

We contribute to theory by advancing the understanding of power dynamics (Hunt et al., 2024; Hurni et al., 2022) in digital platform ecosystems and developing a framework for how power is contested and negotiated during the developmental phase of a regulation, guiding future research and informing practical decision-making. By comparing and contrasting relationships across different ecosystems (app store and search platforms), we offer insights into the key drivers and outcomes of power contestation.

From a practical perspective, we provide insights into the strategies used by platform owners and complementors to shape regulation and provide guidance for regulators, platform owners, and complementors in managing relations and regulatory challenges.

### Contributions to theory

The framework of power contestation in digital platform ecosystems under regulatory scrutiny contributes to the literature on platform power and regulation, as well as the broader literature on digital platforms.

The distribution of power in platform ecosystems is of growing interest in the IS literature. Studies mainly find that platform owners hold a powerful position in the ecosystem and exercise their power to shape it (Hurni et al., 2022; Perrons, 2009). The power of a platform owner in a relational dyad with a complementor is based on the complementor’s dependence on resources that the platform owner controls (Cutolo & Kenney, 2021). Yet, as outlined in the background section, studies on power dynamics between platform owners and complementors remain scarce.

With our findings, we first add to Cutolo and Kenney (2021) and Hunt et al. (2024) by providing rich insights into the interactions between complementors and platform owners as they engage in power contestation. Our work thereby provides empirical validation of the assumption that platform owners are in a strong position of power. However, it also shows how complementors respond, for example, by forming associations and turning to a regulatory body to voice concerns about the platform owners’ potential misuse of power.

Second, we expand previous empirical work on power dynamics from the enterprise software context (Hurni et al., 2022) by studying two Business-to-Consumer (B2C) oriented digital platform ecosystems: app store and search. While the paradox—platform owners needing voluntary contributions from complementors while also controlling them—remains, our findings show that the degree of dependency is shaped by the nature of complements and the characteristics of complementors. In our case, platform owners are generally less reliant on individual complementors due to the fungibility of offerings and intense competition among them. This contrasts with enterprise software ecosystems, where platform owner–complementor relationships are typically more stable and co-invested, making complementors less replaceable (Schreieck et al., 2022). That said, complementor power within B2C ecosystems is not uniform (Asadullah et al., 2023). Larger players such as Spotify can leverage their strong user bases to exert greater influence, while smaller firms have fewer strategic options. High attrition rates among complementors and coordination challenges make collective action difficult, reducing their individual and collective leverage. Furthermore, we show that the regulator’s role is crucial in shaping power contestation, as it acts as a mediator. Both complementors and platform owners use their relationships with the regulator to advocate for either a power shift or the status quo.

Third, we add to recent work on the regulation of digital platform ecosystems (Gleiss et al., 2023; Jacobides & Lianos, 2021; Kircher & Foerderer, 2024; Li & Wang, 2024) by providing insights into how complementors and platform owners use the legislative process to influence regulation. Vice versa, we show that the legislative process, even before the regulation takes effect, impacts the power contestation between complementors and platform owners as the regulator acts as a mediator, providing a stage for these actors to voice concerns, accusations, and suggestions.

We further contribute to the broader literature on digital platform ecosystems, particularly platform governance (Cusumano, 2022; de Reuver, Sørensen, et al., 2018a, 2018b). Our findings offer new insights into how platform owners balance openness and control, not as a fixed design choice but as a contested and dynamic process. While openness is often presented as a way to attract complementors by granting them decision rights and autonomy (Benlian et al., 2015; Ondrus et al., 2015), our study shows how this openness becomes conditional once platform owners gain dominance. Even in seemingly open ecosystems, platform owners impose strict constraints on complementors, who often lack viable alternatives. This leads to ongoing power struggles that shape, and often limit, the practical degree of openness. Our analysis highlights that the ongoing power contestation between platform owners and complementors shapes the ideal degree of openness.

Finally, we add to discussions on value co-creation and value capture in platform ecosystems. It has been shown that platform owners need to enable value co-creation with complementors and share the value within a way that they recoup their investments in the platform and, at the same time, incentivize complementors to continue contributing to the platform (Kim et al., 2016; Schreieck et al., 2021; Tiwana et al., 2010). Our findings show how platform owners aim to maintain their position of power through power contestation and negotiations with regulators, while using that power to extract more value from the ecosystem.

### Contributions to practice

The framework of power contestation has implications for complementors, regulators, and platform owners. Instead of viewing ecosystem dynamics as static, practitioners should recognize that platform power is continuously challenged and reshaped. This dynamic tension, while rooted in the platform–complementor relationship, increasingly plays out through external channels involving regulators. Understanding these dynamics will enable policymakers and regulators to design more robust and adaptable regulatory frameworks that account for these tactics, ensuring that regulations achieve their intended outcomes.

Complementors exercise some form of power, more subtly, through influence, collective action, or appeals to regulators. This power is not necessarily a power of action but a power of influence that can shape the regulatory process in their favor. While their individual influence may be limited, collective action can amplify their voice. Participation in representative associations, for example, offers a structured way of drawing attention to regulations and shaping rules more in line with their interests.

For regulators, our findings imply that platform resistance is not always obvious. Beyond lobbying or legal challenges, platform owners may subtly reinterpret rules or exploit ambiguities to delay or dilute enforcement. Understanding these non-compliance strategies is critical for developing effective regulatory responses. Regulators who recognize subtle tactics like strategic reinterpretation of rules or the intentional creation of compliance ambiguities can better tackle non-compliance and ensure that regulations achieve their intended objectives. These insights can help design more effective compliance monitoring strategies, ensuring strict compliance and tackling any signs of non-compliance or resistance from platform owners. Other practical efforts could involve fostering dialogue mechanisms to mediate these positions and designing regulations that encourage cooperation rather than reinforce opposition.

Platform owners, in turn, may learn that refuting complementor requests and complaints can lead to stricter regulations. They might engage in a more constructive dialogue with complementors to address their concerns instead of involving regulatory bodies. Proactively addressing these concerns could ease tensions and foster an environment in which both sides are less entrenched in their positions, reducing the risk of regulatory escalation. In the long run, this could help platform owners establish more trustful relationships with complementors and retain them even when competing platform ecosystems emerge. Thus, to orchestrate an ecosystem successfully in the long run, it is necessary to optimize elements of platform governance such as openness, control, and boundary resources and manage the power dynamics within the ecosystem.

### Limitations and future research

Our study has several limitations. One limitation is its focus on the European legal area. Although we assume the DMA will have far-reaching effects beyond the European Union (Bradford, 2019), this limitation motivates performing a replicative study for another legislative region. For example, in the US, regulatory actions (Subcommittee on Antitrust, 2020) have progressed with the introduction of the App Store Freedom Act (U.S. Congress 2025), which seeks to enhance competition in mobile app stores by requiring dominant platform owners (such as Google and Apple) to allow sideloading, third-party app stores, and alternative payment options (U.S. Congress, 2025). Analyzing the discourse surrounding this legislative initiative could further corroborate and enhance the findings of this study.

Second, the DMA applies only to a select group of very large “gatekeeper” platforms. Power contestation is also unfolding in smaller ecosystems facing more market competition. How these changes in the context affect the power contestation between platform owners and complementors could be a fruitful avenue for future research.

Third, we have interpreted the digital platform ecosystems we studied as comprising platform owners, complementors, and end users. This interpretation is a simplification because, among others, some complementors are platform owners themselves. For example, Spotify is a complementor in Google’s and Apple’s app store platform ecosystem and, at the same time, the platform owner of its music streaming platform. Relatedly, complementors are often active in several digital platform ecosystems. For example, the platform Trivago maintains relationships with other platforms, such as Google’s ad platform, Meta’s social media platforms, and other travel platforms, such as Booking. These platforms are, in turn, in relationships with each other. Thus, future research could identify the mechanisms and dynamics of the power relations between platform ecosystems. Likely, leaning on power theories, the availability and distribution of resources within and between platforms play a role (Fleming & Spicer, 2014). Additionally, our analysis does not include the regulator’s actions toward platform owners and complementors, but treats them as passive recipients of arguments.

Fourth, while our study focuses on the development phase of the DMA and the observed forms of power contestations within digital platform ecosystems under regulatory scrutiny, future research could examine the post-enforcement phase, where power is exercised less through discourse and more through strategic action. Initial observations suggest that platform owners may engage in circumvention strategies that exploit legal ambiguities (Hart, 1961; Lanamäki et al., 2025; Parker & Nielsen, 2011). Studying these strategic responses, using the European Commission’s ongoing investigations into Alphabet, Apple, and Meta as illustrative cases, could reveal distinct power mechanisms. Such work would extend our findings by exploring how gatekeepers adapt and reassert dominance under regulatory pressure and how these adaptations affect ecosystem governance, complementors, and market contestability.

Finally, the propositions apply to the context of platform ecosystems under regulatory scrutiny. Each outlines testable relationships between key actors (platform owners, complementors, and regulators) and a central outcome: platform owner power. Drawing on prior research that has successfully defined the perceived platform owner power as a measurable construct (Heimburg et al., 2024), future studies could employ survey-based methods to quantitatively assess how advocacy, negotiation, and defensive strategies influence shifts in perceived or actual platform power. This could both validate and extend our conceptual framework toward a more generalizable theory of power contestation in digital platform ecosystems.

## Conclusion

Power contestations between platform owners and complementors will shape the future development of digital platform ecosystems. In this study, we develop a conceptual framework that captures how platform owners and complementors engage in power contestation and strategically interact with a regulator positioned as a mediator. Adding to the current state of IS research on power in and regulation of digital platform ecosystems, we show that regulatory scrutiny impacts the power contestations between complementors and platform owners. By engaging in a legislative process, the regulator provides opportunities for both parties to advocate for either a power shift or maintaining the status quo. Our findings contribute to the literature by highlighting the dynamic and contested nature of power in digital ecosystems and the critical role of regulation in shaping these interactions. We also emphasize the need for platform owners to balance ecosystem governance with complementor relations to navigate regulatory challenges effectively. We hope our work can serve as a starting point for future research on power dynamics within digital platform ecosystems and on the regulator’s role in increasing fairness within them.

## Supplementary Information

Below is the link to the electronic supplementary material.

ESM 1 (DOCX 55.9 KB)

## Data Availability

The data supporting this study's findings are publicly available from the news outlets and sources cited in this article, though access restrictions (including paywalls and copyright limitations) may apply depending on the source. All referenced documents are identified by number in the appendix with source information. The full list of documents is available from the authors upon reasonable request.

## Footnotes

1 Booking.com was later designated as gatekeeper in May 2024.

2 We also searched “Digital Services Act” for the period up to December 2020, as the DMA was only spun off as of then.

3 Abbreviation for Google, Apple, Meta, Microsoft, and Amazon.

4 Sideloading refers to the process of installing apps or transferring files to a device from unofficial sources or channels outside of the official app stores.
