# Supplementary material for: Power contestation and regulation in digital platform ecosystems—The case of the EU’s Digital Markets Act
Source: Electron Mark. 2026 Jan 13;36(1):7. doi: 10.1007/s12525-025-00858-9 (PMC12799660; doi:10.1007/s12525-025-00858-9)
Supplement: Supplementary file 1 — (DOCX 55.9 KB) [file 12525_2025_858_MOESM1_ESM.docx]

# Online Appendix

| **App store platform ecosystem** | | | |
| --- | --- | --- | --- |
| **1^st^-order codes** | **2^nd^-order themes** | **Aggregated dimensions** | **Complementor** |
| - Content censorship and control. - Unfair financial practices - commission and payment issues. - User experience and interface manipulation. - Unfair limitation of innovation and competition. - Restrictive practices by Apple. | Anti-competitive, unfair treatment of complementors | Accusations |  |
| - POs dictate how users install apps under the guise of security. - Privacy/security reason for censoring/blocking innovation and competing products. | Privacy and  security concerns  manipulation |  |  |
| - Gatekeeper bypassing regulations. - Gatekeepers may impose tougher non- financial conditions. - Maintain profit from provisions until legal obligation. | Compliance  avoidance |  |  |
| - Urged to prevent large companies from leveraging their size unfairly. - Push for the same rules to all app stores. | Equality and non-discrimination | Push for Regulation |  |
| - Avoiding shifting abuse. - Block avenues of circumvention. - Ensure timely and strict enforcement. | Strict regulatory  enforcement |  |  |
| - Regulation on payment solutions. - Transparency and consumer protection. - Restrict manipulation practices to favor own platforms. | Regulatory  framework |  |  |
| - PO avoiding gatekeeper status. - Gatekeeper strategies to bypass commission. - Anticipate certain circumvention attitudes. | Potential regulation  circumvention | Concern of  Platform Owner  Behavior |  |
| - Growth of players relies on platforms offering ample support. - Need gatekeepers to protect against piracy and malware for survival. | Risk of unfair  treatment of smaller players |  |  |
| - The possibility to negotiate terms and conditions. - Access to data. - Have a predictable business relationship with gatekeepers. | Active participation and engagement | Demands |  |
| - Fees and access conditions must be fair, reasonable, and non-discriminatory. - Pricing structure should be transparent and unambiguous. | Fair fees and  conditions |  |  |
| - Regulation is inefficient if compliance is not strictly enforced. - Risk of embarrassment if regulation not respected. | Compliance failure warning | Pressure |  |

*Table A.1:* *Coding scheme, following Gioia et al., 2013 – app store platform ecosystem, complementor*

| **App store platform ecosystem** | | | |
| --- | --- | --- | --- |
| **1^st^-order codes** | **2^nd^-order themes** | **Aggregated dimensions** | **Platform owner** |
| - Emphasize willingness to experiment and improve (billing optionality). - Engage with the commission to discuss key aspects of regulation. | Intent to comply | Commitment &  Strategic Support |  |
| - Regulatory attention deflection - strategic support for regulation - directed at a rival | Scrutiny deflection |  |  |
| - Ensures transparency and fairness. - Ensures market competition and neutrality. - Demonstrate a commitment to equity and transparency in dealings with developers. | Transparency and neutrality assertion | Defensive Argumentation |  |
| - Positioning self as a platform that supports innovation and growth within its ecosystem. - Claims significant earnings growth for small app developers. | Innovation and growth support |  |  |
| - Claims fee policy approach aligns with market practices. - Defends the right to choose which app is allowed on the store (in all transparency). | Defends way of operating |  |  |
| - Assures privacy and security for Europeans. - Claims platform integrity crucial for unique features. | Showcasing  consumer  protection efforts |  |  |
| - Warning regulation threatens privacy and security. - Warning of negative impact of the rule on the economy. - Consumer mistrust could harm developers and the app store ecosystem. | Warning of  regulation threats on the market | Warning and Pressure |  |

*Table A.2:* *Coding scheme, following Gioia et al., 2013 – app store platform ecosystem, platform owner*

| **Search platform ecosystem** | | | |
| --- | --- | --- | --- |
| **1^st^-order codes** | **2^nd^-order themes** | **Aggregated dimensions** | **Complementor** |
| - Algorithmic bias and manipulation. - Use of bait and switch model by Google. - Prominence and design bias. - Search result architecture favoring Google results by design. | Deceptive  practices | Accusations |  |
| - Google non-compliant with the DMA rule on preferential treatment. - Current Google One box display is non-compliant. | Regulatory non-  compliance |  |  |
| - Preference for own services and products. - Traffic to merchants and CSS control. - Dominance primarily increases Google’s revenues while raising costs for stakeholders. - Unequal treatment of services. | Market  dominance and  monopoly |  |  |
| - Concern giving gatekeepers full authority to decide ranking criteria. - Economic concern – Google’s bias could harm the job market. - Caution against data misuse. | Concerns about  platform owner  authority | Concern of Platform Owner Behavior |  |
| - Gatekeepers might use strategies to circumvent policy. | Potential policy  evasion |  |  |
| - Push for equal prominence, attractiveness, and functionality. - Advocating for competition mandated by DMA. - Equal treatment and non-discrimination. - Fairness in auction and search interface. | Competition  and equal treatment advocacy | Push for Regulation |  |
| - Evaluating differentiation at the service level. - Gatekeepers must comply with the law. - Reporting unequal treatment. | Regulatory  compliance and  oversight |  |  |
| - Participatory and effective monitoring of compliance with the rules. - Monitoring the impact of regulation on Google. | Compliance  monitoring | Demands |  |
| - Improving user experience. - Re-design for fair competition. - Organic results over proprietary favoritism. | Content improvement and diversity |  |  |
| - Need for equal access to data. - Advocate for ethical boundaries to ensure fair competition. - SEO and auction participation equality. | Advocating for fair  competition &  equal treatment |  |  |

*Table A.3: Coding scheme, following Gioia et al., 2013 – Search platform ecosystem, complementor*

| **Search platform ecosystem** | | | |
| --- | --- | --- | --- |
| **1^st^-order Codes** | **2^nd^-order themes** | **Aggregated dimensions** | **Platform owner** |
| - Fair competition commitment. - Fair process in ranking assertion. - Importance of fair process, not of outcome affirmation. | Fairness and  impartiality  endorsement | Commitment & Strategic Support |  |
| - Pursue innovation with compliance. - Require ongoing dialogue regarding compliance with regulation to address concerns. - Commitment to ongoing engagement with the regulator. - Provide regulator ability to monitor fairness. | Legal compliance  readiness |  |  |
| - Support and deflect scrutiny from self - Support regulation to curb competitor power | Selective endorsement |  |  |
| - Assurance of existing fairness and impartiality. - Regulation criteria clarification demonstrating existing fairness. - Dedication to providing diverse search results and formats. | Fairness &  contestability  assertion | Defensive Argumentation |  |
| - Regulatory compliance with operational flexibility. - Constructing regulatory interpretation defenses. | Strategic rule  interpretation |  |  |
| - Innovation and compliance - change in progress. - Highlight that actions align with the legal framework. - Demonstrate existing compliance through regulation interpretation. | Existing compliance  demonstration |  |  |

*Table A.4: Coding scheme, following Gioia et al., 2013 – Search platform ecosystem, platform owner*

| **Document ID** | **Source Title** | **Source / Publisher** | **Date** | **Source Link** |
| --- | --- | --- | --- | --- |
| D16 | ITI, Experts Analyze DMA in Conversation on the Future of Digital in Europe | Information Technology Industry (ITI) | Dec-20 | <https://www.itic.org/news-events/news-releases/iti-experts-analyze-dma-in-conversation-on-the-future-of-digital-in-europe> |
| D88 | EU travel tech position paper on ex ante regulation to complement competition law in the digital era | EU travel tech | Dec-20 | <https://assets.publishing.service.gov.uk/media/5fce0ec08fa8f54d5e4c530a/Response_to_CFI_-__eu_travel_tech.pdf> |
| D99 | EU travel tech position paper on ex ante regulation to complement competition law in the digital era #2 | EU travel tech | Dec-20 | Available on PoliticoPro |
| D107 | Google pushes back against ex ante rules for gatekeeping platforms | PoliticoPro | Sep-20 | Available on PoliticoPro |
| D543 | EU privacy regulator calls for ban on most targeted ads | PoliticoPro | Feb-21 | Available on PoliticoPro |
| D591 | Morning Tech: Data in the DMA —Antitrust in France—AI in Council | PoliticoPro | Oct-21 | Available on PoliticoPro |
| D625 | Morning Tech: Haugen, round two —Apple vs. Spotify, round two—Commission win over Amazon | PoliticoPro | Nov-21 | Available on PoliticoPro |
| D662 | Search engines see opportunity to lure users away from Google | PoliticoPro | Oct-21 | Available on PoliticoPro |
| D672 | Apple’s Fight for Control Over Apps Moves to Congress and EU | The Wall Street Journal | Jun-21 | <https://www.wsj.com/tech/apples-fight-for-control-over-apps-moves-to-congress-and-eu-11624440601> |
| D676 | Position paper on the Proposal for a regulation on contestable and fair markets in the digital sector | ENPA & EMMA | Sep-21 | <https://www.magazinemedia.eu/storage/103/2021-09_EMMA-ENPA-Position-Paper-on-the-DMA.pdf> |
| D678 | Digital Markets Act Position Paper | The App Association | Mar-21 | <https://actonline.org/wp-content/uploads/ACT-The-App-Association-DMA-Position-Paper-March-.pdf> |
| D679 | Feedback from: Apple | Apple / European Commission | Jun-20 | <https://ec.europa.eu/info/law/better-regulation/have-your-say/initiatives/12417-Digital-Services-Act-deepening-the-internal-market-and-clarifying-responsibilities-for-digital-services/F535714_en> |
| D691 | Reuters Articles “Digital Markets Act” | Reuters | Oct-20 –  Dec-21 | Collection of multiple Reuters articles |
| D722 | DuckDuckGo’s position on the Digital Markets Act | DuckDuckGo | Mar-21 | <https://staticcdn.duckduckgo.com/press/DuckDuckGo-position-on-the-Digital-Markets-Act_March-2021.pdf> |
| D746 | Consultation on the European Commission’s Inception Impact Assessment | Google / European Commision | Jun-20 | <https://ec.europa.eu/info/law/better-regulation/have-your-say/initiatives/12416-Single-Market-new-complementary-tool-to-strengthen-competition-enforcement/F535558_en> |
| D787 | Position paper on the Digital Markets Act (DMA) | Digital SME | Apr-21 | <https://www.digitalsme.eu/digital/uploads/Position-paper-on-Digital-Markets-Act-FINAL-DRAFT.pdf> |
| D803 | News Media Europe position paper on the European Commission proposal for a Digital Markets Act (DMA) | News Media Europe | Apr-21 | <https://www.newsmediaeurope.eu/wp-content/uploads/2021/04/News-Media-Europe-position-Digital-Markets-Act-proposal-April-2021.pdf> |
| D808 | DIGITAL MARKETS ACT PROPOSAL Position Paper | BEUC | Apr-21 | <https://www.beuc.eu/sites/default/files/publications/beuc-x-2021-030_digital_markets_act_proposal.pdf> |
| D837 | Digital Markets Act: The Need For a More Targeted and More Efficient Tool | EU travel tech | Feb-21 | <https://eutraveltech.eu/digital-markets-act-the-need-for-a-more-targeted-and-more-efficient-tool/> |
| D906 | Developers Alliance Standpoints On The Digital Services Act And The Digital Markets Act | Developers Alliance | Oct-19 | <https://developersalliance.org/developers-alliance-standpoints-on-the-digital-services-act-and-the-digital-markets-act/> |
| D925 | Google in last-ditch lobbying attempt to influence incoming EU tech rules | Financial Times | Jan-22 | <https://www.ft.com/content/8c7527bc-7ab4-41cd-ba94-3145208da9c3> |
| D1011 | Feedback from: Yelp | Yelp |  |  |
| D1041 | Statement on the European Parliament plenary vote on the Digital Markets Act and the Digital Services Act | The App Association | May-22 | <https://actonline.org/statements/page/3/> |
| D1045 | EU pries open Apple’s grip on payment tech | PoliticoPro | Mar-20 | Available on PoliticoPro |
| D1059 | Digital Brief: DMA Digital Brief: DMA final text, DSA approaching, AIA draft report final text, DSA approaching, AIA draft report | Euractiv | Apr-22 | <https://www.euractiv.com/section/tech/news/digital-brief-powered-by-greens-efa-dma-final-text-dsa-approaching-aia-draft-report/> |
| D1087 | Feedback from: Apple | Apple / European Commission | Jun-20 | <https://ec.europa.eu/info/law/better-regulation/have-your-say/initiatives/12418-Digital-Services-Act-package-ex-ante-regulatory-instrument-of-very-large-online-platforms-acting-as-gatekeepers/F535696_en> |
| D1118 | EU travel tech welcomes the DMA agreement and stresses that enforcement and complementarity with EU competition law will remain crucial | EU travel tech | Mar-22 | <https://eutraveltech.eu/press-release-eu-travel-tech-welcomes-the-dma-agreement-and-stresses-that-enforcement-and-complementarity-with-eu-competition-law-will-remain-crucial/> |
| D1137 | Apple’s Proposed Changes Reject the Goals of the DMA | Spotify | Jan-24 | [https://newsroom.spotify.com/2024-01-26/apples-proposed-changes-reject-the-goals-of-the-dma/#:~:text=For%20almost%20five%20years%20%E2%80%93%201%2C782,like%20Apple%20to%20go%20unchecked.](https://newsroom.spotify.com/2024-01-26/apples-proposed-changes-reject-the-goals-of-the-dma/#:~:text=For%20almost%20five%20years%20%E2%80%93%201%2C782,like%20Apple%20to%20go%20unchecked.) |
| D1138 | The DMA Means a Better Spotify for Artists, Creators, and You | Spotify | Jan-24 | <https://newsroom.spotify.com/2024-01-24/the-dma-means-a-better-spotify-for-artists-creators-and-you/> |
| D1139 | A Letter to the European Commission on Apple’s Lack of DMA Compliance | Spotify | Mar-24 | <https://newsroom.spotify.com/2024-03-01/a-letter-to-the-european-commission-on-apples-lack-of-dma-compliance/> |

*Table A.5: Document references (the complete dataset is available upon request)*
